# A Proposal for Modest Revision of the Definition of Type 1 and Type 2 Myocardial Infarction

**DOI:** 10.1161/CIRCULATIONAHA.119.042157

**Published:** 2019-09-06

**Authors:** James A. de Lemos, L. Kristin Newby, Nicholas L. Mills

**Affiliations:** 1 Division of Cardiology, Department of Medicine, University of Texas Southwestern Medical Center, Dallas (J.A.d.L.).; 2 Division of Cardiology, Department of Medicine, Duke Clinical Research Institute, Duke University Medical Center, Durham, NC (L.K.N.).; 3 British Heart Foundation Centre for Cardiovascular Science and Usher Institute of Population Health Sciences and Informatics, University of Edinburgh, United Kingdom (N.L.M.).

**Keywords:** anemia, coronary artery disease, hypertension, myocardial infarction, tachyarrhythmia

The Universal Definition of Myocardial Infarction (UDMI)^1^ classifies myocardial infarction (MI) into 5 subtypes, of which type 1 and type 2 MI are the most common and relevant to practicing clinicians. Type 1 MI is defined as MI caused by acute atherothrombotic mechanisms, with type 2 MI defined as MI resulting from myocardial oxygen supply/demand mismatch without acute atherothrombosis. The UDMI recognizes multiple potential causes of type 2 MI, including demand-side abnormalities such as tachyarrhythmia or severe hypertension, and supply-side issues such as severe anemia, hypoxemia, or hypotension. Type 2 MI may occur with or without obstructive coronary disease, with the threshold for type 2 MI lower in patients with fixed obstructive coronary artery disease.

Type 2 MI is common and is associated with substantial risk for cardiac (and noncardiac) death and major adverse cardiac events.^2^ Research to date has been limited largely to observational studies that have used varying definitions and adjudication criteria for type 2 MI, focusing on prevalence, risk factors, and prognosis, with almost no data on treatment.^2^ Recently, an *International Classification of Diseases, Tenth Revision* (ICD-10) code was introduced for type 2 MI, and it is hoped that this will facilitate research using administrative data.^3^ However, we believe that the current definition for type 2 MI is too phenotypically heterogeneous to permit adequate study or reliable coding by hospital administrators.

It is important to note that the UDMI includes, under the umbrella of type 2 MI, several acute coronary processes that obstruct blood flow, including spontaneous coronary artery dissection (SCAD), coronary embolism, and coronary vasospasm. We believe that these specific diagnoses are a poor fit in the category of type 2 MI; from both a pathophysiological and clinical perspective, they are more closely aligned with type 1 MI. SCAD, coronary embolism, and vasospasm are acute supply-side obstructive processes that have clinical presentations and initial diagnostic and management approaches that are similar to type 1 MI. They are usually spontaneous presentations, without an obvious precipitating event. These conditions typically are initially triaged as suspected acute coronary syndromes, treated with guideline-recommended therapies for acute coronary syndromes, and evaluated with early coronary imaging. The diagnosis is usually made in the cardiac catheterization laboratory, with subsequent treatment determined by findings from coronary imaging. In contrast, most other etiologies causing type 2 MI, including severe tachycardia and hypertension, anemia, and hypoxemia, are apparent at the time of clinical presentation, and diagnosed based on clinical criteria, with coronary angiography delayed or deferred.

Applying the same diagnosis of type 2 MI to such phenotypically distinct patients has clear disadvantages for clinical management, and negatively impacts the quality of research into type 2 MI. Reporting the epidemiology, outcomes, and treatment responses of type 2 MI, as currently defined, is of little value other than making sure that these diagnoses do not muddy the interpretation of type 1 MI. However, including patients without acute atherothrombosis in the type 1 MI category also creates problems. Clinical trials and guideline recommendations for management of acute coronary syndromes are only applicable to type 1 MI. For example, applying therapies tested in atherothrombosis, such as parenteral anticoagulation and intracoronary stenting, to patients with SCAD may be harmful.^4^ Evidenced-based therapies exist for coronary vasospasm and are emerging for SCAD, with coronary embolism typically managed empirically based on the source of embolism. However, such therapies are clearly distinct from those used to treat acute atherothrombosis.

We propose consideration of a modest redefinition of type 1 and type 2 MI (Figure), with type 1 MI defined by acute coronary obstruction or reduction in coronary blood flow rather than by atherothrombosis. This would move SCAD, coronary embolism, and coronary vasomotor abnormalities (including epicardial vasospasm and microvascular dysfunction) into the type 1 MI category. We further propose subclassifying type 1 MI based on the underlying pathophysiology, with type 1A MI being the typical atherothrombosis category and the other etiologies having separate subclassifications (Figure). Type 2 MI would be defined as MI attributable to acute supply/demand mismatch without acute coronary obstruction. We propose further subclassifying type 2 MI into those with or without obstructive coronary artery disease (Figure), because the subsequent management approaches differ substantially based on the presence of severe coronary artery disease. As an important corollary, modification of *International Classification of Diseases*(ICD) codes to improve specificity would be an important step forward for research and quality improvement in patients with MI caused by factors other than atherothrombosis.

**Figure.**
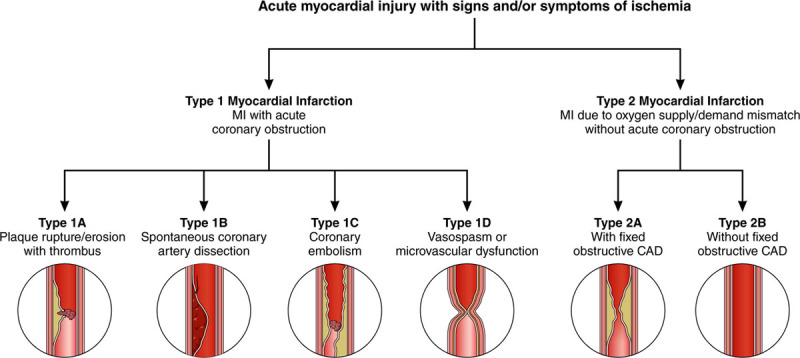
**Proposed revision to Universal Definition of Myocardial Infarction (UDMI).** The proposal redefines type 1 and type 2 MI based on the presence or absence of acute coronary obstruction, with subclassifications based on underlying pathophysiology. This differs from the current UDMI by categorizing spontaneous coronary dissection, coronary embolism, and vasospasm as type 1 MI, and subclassifying type 2 MI based on the presence or absence of fixed obstructive coronary disease. CAD indicates coronary artery disease; and MI, myocardial infarction.

We believe that this redefinition would better align with modern approaches to diagnosis and management of the spectrum of patients with MI. It would facilitate research into specific diagnostic subcategories and identification of optimal treatment approaches. This designation would also eliminate the need for a separate classification scheme for myocardial infarction with no obstructive coronary disease.^5^ Each of the categories of myocardial infarction with no obstructive coronary disease would fit within the new MI definition construct. Also, the creation of subcategories (type 1A, 1B, etc) would allow room for additional MI phenotypes, as new information on pathophysiology becomes available, without altering the fundamental structure of the classification.

Although some may view differences between the Universal Definition of Myocardial Infarction and our proposed revision as little more than administrative details, we would argue that alignment of diagnosis with clinical presentation, pathophysiology, and diagnostic approach is an essential step to address current knowledge gaps. Moreover, accurate diagnosis has direct implications for quality reporting, because evidence-based standards exist only for MI attributable to atherothrombosis. Clinicians should be held accountable only for adhering to process and performance measures for those patients in whom the measures apply. Finally, and arguably most importantly, as we enter the precision medicine era, it is imperative that our diagnoses be as precise as possible.

## Disclosures

Dr de Lemos has received grant support from Roche Diagnostics and Abbott Diagnostics and consulting income from Roche Diagnostics, Abbott Diagnostics, Ortho Clinical Diagnostics, Siemens Health Care Diagnostics, Radiometer and Quidel Cardiovascular, Inc. Dr Newby has received consulting or Advisory Board fees from Ortho Clinical Diagnostics, Roche Diagnostics, and Metanomics, outside the submitted work. Dr Mills has received honoraria or consulted for Abbott Diagnostics, Siemens Healthineers, Singulex, and LumiraDx and has received research support from the British Heart Foundation through the Butler Senior Clinical Research Fellowship (FS/16/14/32023). The University of Edinburgh has received research grant funding from Abbott Diagnostics and Siemens Healthineers.
